# Vegetation Structure Drives Seasonal and Diel Dynamics of Avian Soundscapes in an Urban Wetland

**DOI:** 10.3390/plants15071023

**Published:** 2026-03-26

**Authors:** Zhe Wen, Zhewen Ye, Yunfeng Yang, Yao Xiong

**Affiliations:** 1 College of Landscape Architecture, Nanjing Forestry University, No. 159 Longpan Road, Xuanwu District, Nanjing 210037, China; wenzhe@njfu.edu.cn (Z.W.); zwyeh@njfu.edu.cn (Z.Y.); 2 College of Art and Design, Nanjing Forestry University, No. 159 Longpan Road, Xuanwu District, Nanjing 210037, China; emmabear@njfu.edu.cn

**Keywords:** urban wetland, soundscape ecology, vegetation structure, acoustic indices, anthropogenic disturbance, seasonality

## Abstract

Urban wetlands are acoustic hotspots where vegetation structure, hydrological dynamics, and anthropogenic noise interact, yet multi-season assessments of how vegetation influences avian soundscapes are limited. This study explored bird soundscape dynamics across forest, open forest grassland, and meadow habitats in Nanjing Xinjizhou National Wetland Park, eastern China, using passive acoustic monitoring during spring and autumn 2023. Twelve sampling points (four per vegetation type) were established, and six acoustic indices were calculated, including the Acoustic Complexity Index (ACI), Acoustic Diversity Index (ADI), Acoustic Evenness Index (AEI), Bioacoustic Index (BIO), Normalized Difference Soundscape Index (NDSI), and Acoustic Entropy Index (H). were calculated from 48-h recordings each season. Random forest models and redundancy analysis assessed the relationships between acoustic indices, fine-scale vegetation parameters (e.g., crown width, tree height, species richness), and anthropogenic factors (e.g., distance to roads/trails, surface hardness). Vegetation structure, particularly crown width, was the primary driver of avian acoustic diversity, with broad-crowned forests consistently exhibiting the highest acoustic complexity. In spring, anthropogenic factors such as trail and road proximity dominated soundscape variation, suppressing biological sounds. In autumn, with reduced human presence, vegetation structure emerged as the dominant factor, while bioacoustic activity remained elevated despite reduced peaks in acoustic complexity. Proximity to roads increased low-frequency (1–2 kHz) noise and suppressed mid-frequency (4–8 kHz) bird vocalizations, but trees with crown widths ≥4 m maintained higher acoustic diversity even near disturbance sources. This study demonstrates that vegetation structure mediates both resource availability and sound propagation, buffering the effects of anthropogenic disturbance in frequency-specific ways. Multi-season sampling is crucial for understanding the dynamic interplay between vegetation phenology and human activity that shapes urban wetland soundscapes.

## 1. Introduction

As urbanization reshapes landscapes worldwide, urban green areas play a critical role in reconciling biodiversity conservation with human well-being [1], with vegetation structure—comprising trees, shrubs, and herbs—forming the main framework for living spaces. It forms the basic framework that affects habitat quality, microclimate, and species interactions. A good way for people to understand the ecological processes at play is soundscape ecology, which uses sounds in the environment in an attempt to monitor changes in an ecosystem without interfering with it [2]. Urban wetlands, in particular, serve as acoustic hotspots where the structure of vegetation interacts with hydrodynamic forces and human-made noise, creating ideal environments for studying how vegetation influences biological soundscapes [3].

At the core of soundscape ecology are acoustic indices—quantitative metrics that simplify complex sound data into measurable parameters by analyzing frequency, intensity, and temporal patterns [4]. These indices, including the Acoustic Complexity Index (ACI), Acoustic Diversity Index (ADI), and Normalized Difference Soundscape Index (NDSI), have been positively linked to bird species richness in temperate habitats [5]. The ACI, for instance, measures the complexity of soundscapes by analyzing changes in acoustic signal structure [6,7], while the ADI quantifies the diversity of sound sources in a given area. The NDSI, on the other hand, compares the acoustic characteristics of different environments and is particularly useful for assessing anthropogenic disturbance levels in soundscapes, providing insights into habitat suitability for wildlife, especially when the acoustic requirements of a species are known [8]. These indices offer a scalable approach for connecting measurable vegetation characteristics—such as tree height, canopy width, and foliage density—with biological responses. By linking acoustic features to the structural attributes of vegetation, these indices overcome some of the limitations of traditional ecological methods, offering a non-invasive way to assess how vegetation structure supports avian communities. This non-invasive approach makes it possible to monitor and manage biodiversity in urban and natural ecosystems more effectively.

Among the biological components of urban soundscapes, birds are the primary acoustic mediators of the sound environment [9]. Their vocalizations dominate the biophony of many terrestrial ecosystems, particularly during the dawn and dusk choruses [2,10]. Birds are particularly valuable for soundscape studies because they are highly sensitive to vegetation structure. They rely on trees not only as singing perches but also for nesting and foraging. In addition, birds respond quickly to anthropogenic disturbances, such as noise and habitat fragmentation, making them effective indicators of environmental changes [11]. Another key feature of birds is that their vocalizations span a broad frequency range (2–11 kHz), which typically avoids overlap with the low-frequency noise from traffic (1–2 kHz) [12], though some wetland birds may vocalize in this lower frequency range. This distinction facilitates the clear separation of bird calls from human-made noise, enabling more accurate soundscape analyses [13]. Therefore, bird soundscapes serve as an integrated measure of both habitat quality, mediated by vegetation, and disturbance intensity, shaped by human activity.

Vegetation structure influences bird soundscapes through two complementary mechanisms. First, it determines resource availability. Taller trees and wider canopies provide more singing perches, nesting sites, and foraging opportunities, which support higher densities of vocalizing birds [14]. These resources vary seasonally, and birds adjust their vocalization patterns in response to the changing availability of vegetation-based resources throughout the year. Second, vegetation modulates sound propagation. Dense foliage can attenuate, diffract, and reverberate sound waves, creating frequency-specific filters that may favor certain vocalizations over others [15,16]. During the growing season, denser vegetation helps buffer anthropogenic noise, creating quieter, more favorable conditions for bird vocalizations. However, in the winter months, when vegetation becomes sparse, its reduced buffering capacity exposes birds to higher levels of noise, potentially altering their vocal behavior. For example, natural secondary forests exhibit higher ACI and ADI values than planted forests or meadows, due to their greater vertical heterogeneity and structural complexity [17]. In contrast, open habitats, such as grasslands, are more susceptible to road noise infiltration, which reduces the biophony-to-technophony ratio [18]. These dynamics highlight the importance of maintaining structurally diverse vegetation in urban wetlands, where vegetation not only supports higher bird densities but also mitigates noise pollution, helping preserve the quality of avian soundscapes. Recent studies have shown that vegetation structural complexity not only enhances the representation of bioacoustic signals (ACI/ADI) but also suppresses anthropogenic noise through canopy stratification, demonstrating how vertical heterogeneity shapes acoustic niches in urban ecosystems [19,20]. The interaction between vegetation structure and seasonal or diel vocalization patterns underscores the critical role of vegetation in shaping the acoustic environment of urban wetlands and supporting biodiversity.

Despite recent advances in soundscape ecology, several research gaps remain. A major limitation is that most studies focus on a single season, failing to capture how phenological changes—such as leaf emergence, flowering, and senescence—affect vegetation structure and, in turn, soundscape characteristics throughout the annual cycle [21,22]. For instance, although Hao et al. (2024) showed significant vegetation effects on urban forest soundscapes, their single-season design limits our understanding of how these effects vary across different seasons [23]. Another issue is the use of broad vegetation classifications in soundscape research, such as “forest” versus “grassland,” which overlook important structural details like canopy width, tree height, and vertical stratification. These features are essential for shaping the acoustic environment and should not be overlooked when analyzing soundscape composition [24]. Additionally, wetlands—though the acoustically richest ecosystems on Earth—remain among the least studied in soundscape ecology. Wetland soundscapes uniquely combine biophony from waterbirds, geophony from flowing water, and technophony from surrounding human activity. Water itself produces sound through turbulence and bubble entrainment, with frequencies (0.4–2 kHz) that can overlap with and mask bird vocalizations [25]. However, studies examining how vegetation structure influences bird soundscapes in urbanizing wetlands—where natural and human-made sounds increasingly overlap—are still limited.

To address these gaps, this study explores how vegetation structure influences avian soundscape dynamics in urban wetlands. Specifically, the study seeks to answer the following questions: (1) How do bird soundscapes vary across different vegetation types, such as forests, open forest grasslands, and meadows? (2) What is the relative importance of fine-scale vegetation features—like tree height, canopy width, and species richness—compared to anthropogenic disturbances, such as distance to roads and trails, in shaping acoustic indices? (3) How do these relationships change between spring and autumn, reflecting phenological differences in vegetation and bird behavior? This multi-season, multi-vegetation approach is among the first to separate the combined effects of vegetation structure and anthropogenic disturbance on bird soundscapes in rapidly urbanizing wetlands. The results aim to guide science-based vegetation management strategies that support biodiversity conservation and enhance soundscape design in urban wetlands.

## 2. Results

### 2.1. Spatial and Temporal Characteristics of Bird Soundscapes

#### 2.1.1. Diurnal Variation in Bird Soundscape Index Across Vegetation Types

**Figure 1:**
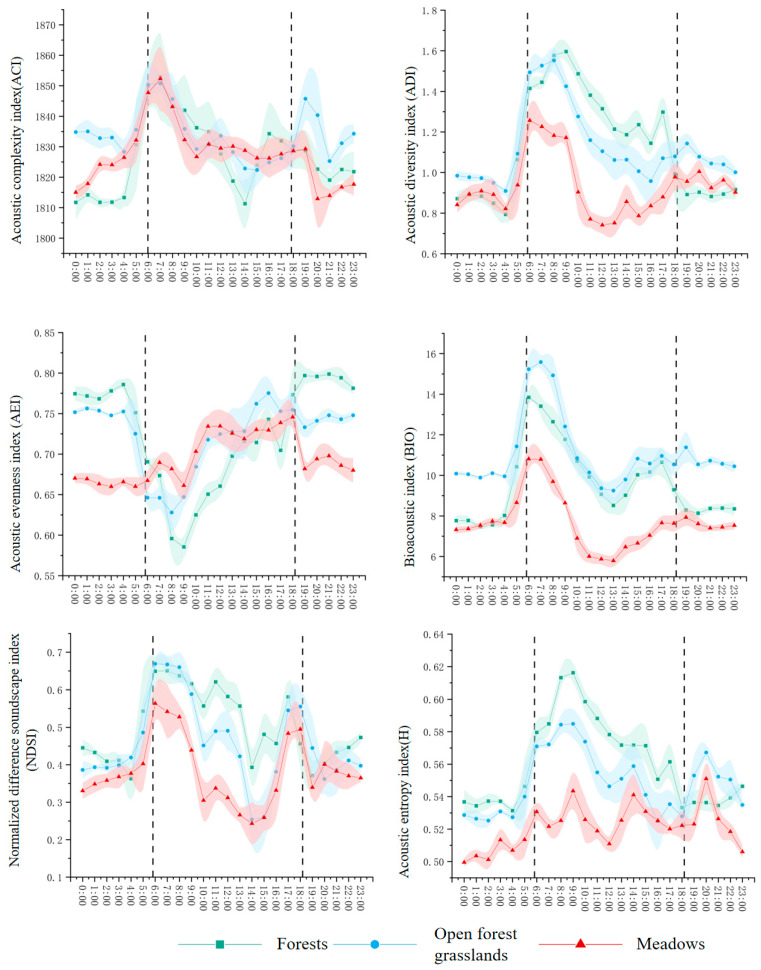
The soundscape indices of different vegetation types vary in day and night (the black vertical line indicates sunrise and sunset times). Black dashed vertical lines indicate sunrise and sunset times. Shaded areas represent the 95% confidence interval of the mean values.

Clear diurnal patterns emerged across all vegetation types, with pronounced peaks in acoustic activity during dawn (05:00–07:00) and dusk (18:00–20:00) (Figure 1). The magnitude and timing of these peaks varied systematically with vegetation structure.

During dawn and dusk, forests exhibited the highest acoustic activity across multiple indices. Acoustic Complexity Index (ACI) and Acoustic Diversity Index (ADI) peaked in forests (ACI: 1.8–2.0; ADI: 0.9–1.1), followed by open forest grasslands (ACI: 1.6–1.8; ADI: 0.8–1.0) (Figure 1). Acoustic Evenness Index (AEI) was also highest in forests (0.75–0.85), suggesting balanced contributions from multiple vocalizing species, while open forest grasslands (0.70–0.80) and meadows (0.65–0.75) showed progressively lower evenness. Normalized Difference Soundscape Index (NDSI) reached its highest values during these periods (forests: 0.6–0.7; open forest grasslands: 0.5–0.6), indicating strong biophony dominance over anthropogenic noise.

During daytime hours (08:00–16:00), acoustic activity generally declined but maintained vegetation-dependent gradients. Forests retained elevated ACI (1.2–1.4) and ADI (0.9–1.1) compared to open forest grasslands (ACI: 1.1–1.3; ADI: 0.8–1.0) and meadows (ACI: 0.9–1.1; ADI: 0.7–0.9) (Figure 1). Acoustic Entropy Index (H) gradually increased from dawn to mid-morning in forests (0.85–0.90), followed by a midday decline and secondary afternoon rise, with forests consistently showing higher H than open forest grasslands and meadows throughout.

During nighttime (20:00–04:00), all indices decreased substantially across habitats. ACI fell to 0.6–0.8 and ADI to 0.5–0.7, indicating minimal nocturnal acoustic activity and reduced sound source diversity (Figure 1). However, Bioacoustic Index (BIO) remained relatively elevated in open forest grasslands (8–10 at dusk, declining but still detectable at night), suggesting sustained biological activity in this transitional habitat. NDSI generally decreased below 0.35 across most habitats, though forests retained higher values (0.4–0.5), indicating a persistent biological sound presence.

These diurnal patterns demonstrate that vegetation structure mediates both the timing and intensity of acoustic activity: structurally complex forests support higher and more diverse vocalizations during peak acoustic periods, while simpler habitats like meadows show attenuated profiles with less pronounced diurnal differentiation.

#### 2.1.2. Seasonal Variation Across Vegetation Types

**Figure 2:**
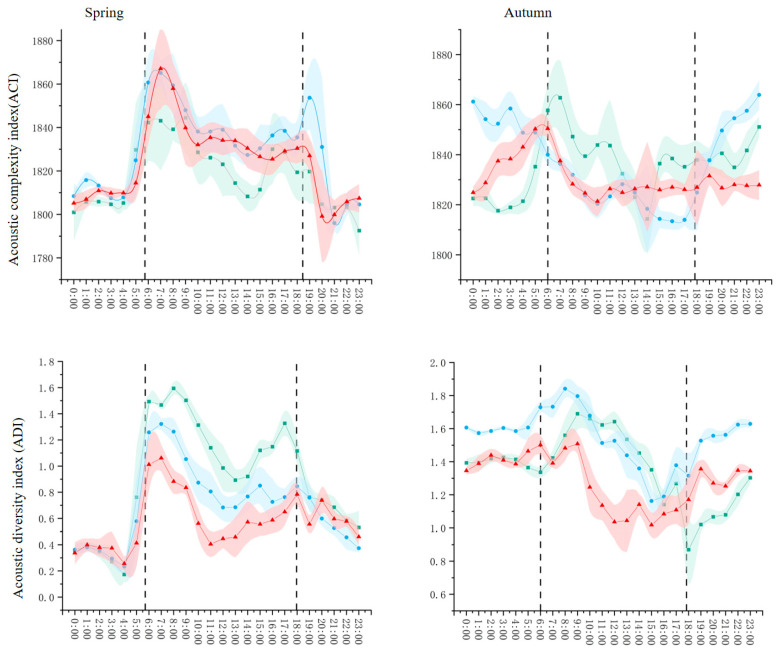

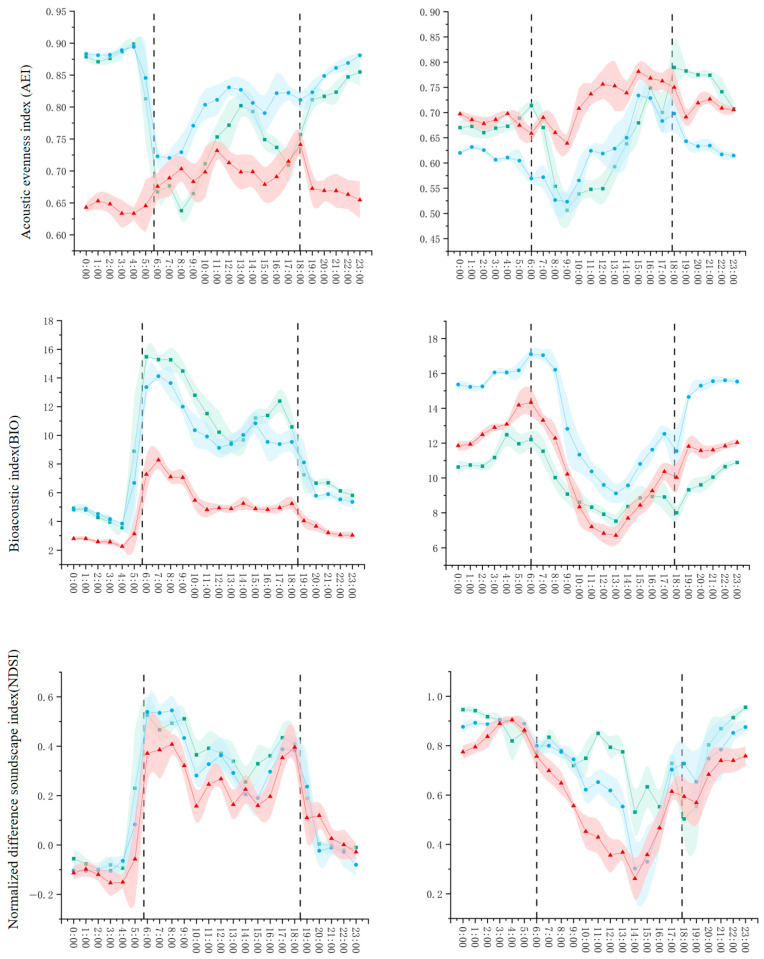

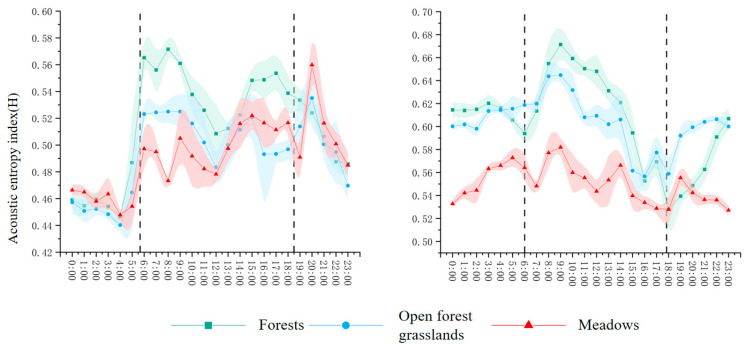
The soundscape indices of different vegetation types vary in spring and autumn. Black dashed vertical lines indicate sunrise and sunset times. Shaded areas represent the 95% confidence interval of the mean values.

Substantial seasonal differences emerged across all acoustic indices and vegetation types, with spring and autumn exhibiting distinct acoustic signatures (Figure 2).

In spring, vegetation types showed pronounced crepuscular peaks in ACI, ADI, AEI, and BIO, with forests having the highest values across most indices, indicating peak breeding-season vocal activity. In contrast, autumn exhibited attenuated crepuscular peaks and more uniform diurnal trajectories. ACI and ADI values were lower, particularly during dawn/dusk, while BIO and NDSI were higher, reflecting post-breeding vocal behavior and reduced anthropogenic noise interference. These seasonal patterns suggest that phenological changes in vegetation and bird behavior jointly shape soundscape dynamics, with spring peaks corresponding to peak breeding-season vocal activity, and autumn reflecting changes in vocal behavior due to post-breeding and altered vegetation structure.

#### 2.1.3. Wind Conditions During Sampling

Throughout the two sampling sessions, wind speeds remained consistently low. During the spring session, wind speeds ranged from 0.5 to 4.2 m/s, with a mean of 1.8 m/s (±0.9 SD); during the autumn session, wind speeds ranged from 0.3 to 3.8 m/s, with a mean of 1.5 m/s (±0.8 SD). Overall, 92% of recording hours experienced wind speeds below 3 m/s, and no recordings exceeded the pre-defined threshold of 5 m/s.

Visual inspection of the 1–2 kHz noise band power showed no apparent correspondence with wind speed fluctuations. Periods of slightly elevated wind speed (e.g., >3 m/s) did not coincide with increases in low-frequency noise, suggesting that the observed noise level variations were primarily driven by anthropogenic activity rather than wind. Given the consistently low wind conditions, wind-induced acoustic interference was considered negligible in this study.

### 2.2. Factors Influencing Bird Soundscape Variation

#### 2.2.1. Random Forest Analysis

**Figure 3:**
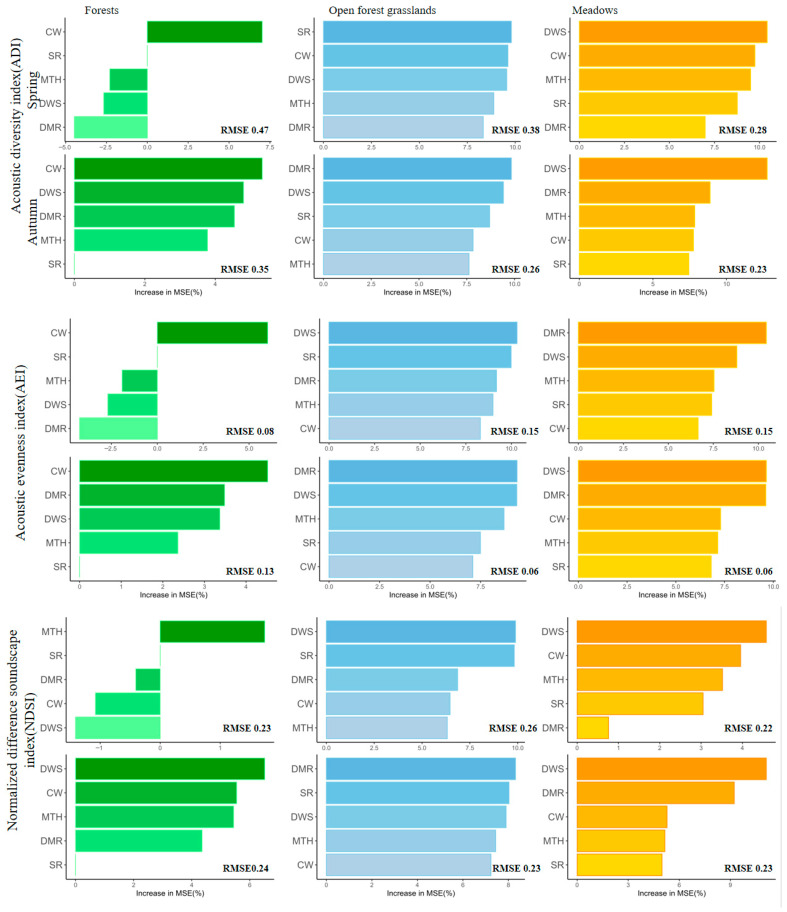
Random forest analysis of the impact of influencing factors on the soundscape indices (SR: species richness; MTH: mean tree height; DWS: distance from water source; DMR: distance from main road; CW: crown width). Darker colors indicate a greater increase in MSE, representing a stronger influence on the soundscape indices.

Random forest models explained substantial variance in acoustic indices across all vegetation types and seasons (RMSE < 0.05 for ADI, AEI, and NDSI; RMSE < 0.08 for ACI, BIO, and H), indicating good predictive performance. Variable importance varied markedly between seasons and vegetation types (Figure 3).

These results reveal seasonally shifting drivers of acoustic diversity. In forests, vegetation structure (particularly crown width, CW) consistently influenced soundscapes across both seasons, while in open forest grasslands and meadows, anthropogenic factors (such as distance to main road, DMR, and distance to water source, DWS) became increasingly important. Specifically, in spring, CW was a key predictor for ADI and AEI in forests, while DMR strongly influenced ADI and AEI in open forest grasslands. In autumn, DMR and DWS emerged as dominant predictors in open forest grasslands and meadows, respectively. These patterns highlight the dynamic interplay between vegetation structure and human disturbances in shaping soundscape diversity.

#### 2.2.2. Redundancy Analysis

**Figure 4:**
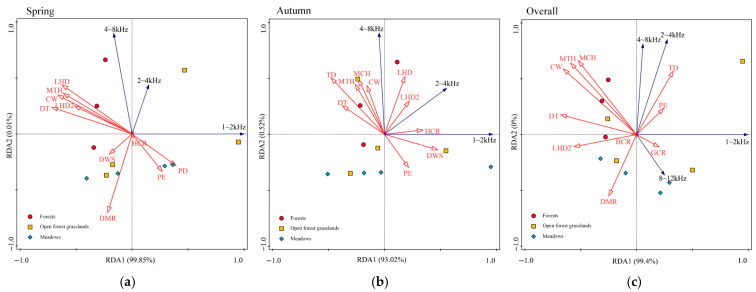
RDA analysis of the impact of influencing factors on soundscape power: (**a**) results in spring; (**b**) results in autumn; (**c**) overall results.

RDA revealed distinct seasonal patterns in the relationships between soundscape frequency composition and environmental gradients (Figure 4, Table 1). The first two RDA axes explained 68.3% of the variance in spring, 59.7% in autumn, and 71.2% in the pooled analysis. The results suggest that vegetation structure and anthropogenic factors influence soundscape diversity, with distinct seasonal shifts in their relative importance across habitats.

Figure 4 presents the results of redundancy analysis (RDA) for spring, autumn, and overall acoustic data, showing how various environmental variables (e.g., crown width, tree density, distance to main road, etc.) influence soundscape power. In each panel, the points represent sampling locations across different vegetation types (forests, open forest grasslands, and meadows), with the four points in the overall panel representing the combined seasonal data. The overlap of points between spring and autumn indicates seasonal variation in the influence of environmental variables, and the direction of vectors reflects the strength and nature of the relationship between these variables and soundscape diversity.

In spring, bird soundscape characteristics were primarily influenced by distance to trail (DT) (45.9% explained, *p* = 0.014), green space ratio (GCR) (36.6%, *p* = 0.006), and distance to main road (DMR) (8.1%, *p* = 0.030) (Figure 4a, Table 1). DT and DMR showed strong negative correlations with normalized power spectral density (nPSD) in the 1–2 kHz band (anthropogenic noise), indicating that greater distance from trails and roads reduces low-frequency anthropogenic sound energy. Conversely, GCR was positively associated with nPSD in the 2–4 kHz band (low-frequency bird vocalizations), reflecting enhanced biological sound in areas with higher vegetation cover. DT and DMR also showed positive associations with nPSD in the 4–8 kHz band (mid-frequency bird vocalizations), suggesting reduced anthropogenic interference allows fuller expression of the avian frequency spectrum.

In autumn, soundscape characteristics were primarily influenced by crown width (CW) (22.2% explained, *p* = 0.014), tree density (TD) (21.2%, *p* = 0.120), hardness rate (HCR) (16.0%, *p* = 0.128), and distance to trail (DT) (15.4%, *p* = 0.186) (Figure 4b, Table 1). CW showed a strong positive association with nPSD in the 4–8 kHz band, indicating that wider canopies support higher mid-frequency bird vocal activity in autumn. HCR was negatively associated with nPSD in the 2–4 kHz band, suggesting that impervious surfaces suppress low-frequency biological sounds. Notably, anthropogenic variables (DT, DMR) explained less variance in autumn than spring, while vegetation structure variables (CW, TD) gained importance.

In the pooled analysis across both seasons, the primary gradients were DT (51.5% explained, *p* = 0.010), HCR (32.1%, *p* = 0.008), and DMR (9.1%, *p* = 0.016) (Figure 4c, Table 1), confirming the overarching importance of anthropogenic disturbance gradients in structuring soundscape frequency composition. However, the seasonal shift in variable importance—from anthropogenic dominance in spring to vegetation structure dominance in autumn—reveals a dynamic interplay between human disturbance and habitat structure across the annual cycle.

These RDA results demonstrate that anthropogenic disturbance (trails, roads, impervious surfaces) suppresses biological sounds, while vegetation structure (crown width, tree density) promotes them, with the relative strength of these effects shifting seasonally as vegetation phenology alters both habitat structure and its sound-buffering capacity.

#### 2.2.3. Relationship Between Noise Levels and Bird Vocalizations

The RDA results presented above (Section 2.2.2, Figure 4) provide quantitative evidence for the relationship between anthropogenic noise and bird vocal activity. Across all vegetation types, NDSI values were consistently lowest during daytime hours (08:00–16:00) and highest during dawn (05:00–07:00) and dusk (18:00–20:00) (Figure 1), reflecting the dominance of low-frequency anthropogenic noise during peak human activity hours. This pattern was most pronounced in open habitats, consistent with the strong negative correlations observed between distance to roads/trails and 1–2 kHz band power. In forests, where vegetation buffers noise intrusion, the diurnal NDSI fluctuation was attenuated.

## 3. Discussion

This study uses passive acoustic monitoring, random forest models, and redundancy analysis to explore the nonlinear effects of environmental variables on six acoustic indices (ACI, ADI, AEI, BIO, NDSI, H) and soundscape power spectral density. By employing a multi-season, multi-vegetation approach, we are able to separate the effects of vegetation structure and anthropogenic disturbance on bird soundscapes in urbanizing wetlands, providing valuable insights into how these factors collectively shape avian soundscapes.

### 3.1. Vegetation Structure Shapes Bird Soundscapes Through Resource Provision and Sound Propagation

Our results demonstrate that vegetation structure, particularly crown width (CW) and tree height (MTH), is the primary driver of bird soundscape variation in forests, with Acoustic Complexity Index (ACI) and Acoustic Diversity Index (ADI) showing strong positive associations with these structural attributes (Section 2.2.1, Figure 3). This finding aligns with previous studies showing that structurally complex forests support higher avian acoustic diversity [23,26]. However, our study extends these observations by revealing that vegetation influences soundscapes through two complementary mechanisms: resource provision and sound propagation.

First, vegetation structure determines resource availability for vocalizing birds. Wider crowns and taller trees provide more horizontal singing perches, nesting substrates, and foraging opportunities, supporting higher densities of acoustically active individuals [14]. Dense canopies also enhance arthropod biomass, supplying food for insectivorous songbirds [27]. These resource-mediated effects were most evident in forests, where CW emerged as the dominant predictor across multiple acoustic indices in both spring and autumn (Figure 3). Second, vegetation modulates sound propagation through physical interactions with acoustic signals [15,16]. Dense foliage can attenuate, diffract, and reverberate sound waves, creating frequency-specific transmission filters that may favor certain vocalizations over others. During the growing season, when vegetation is fully leafed, this sound-buffering capacity is maximized, potentially reducing anthropogenic noise infiltration and creating quieter microhabitats that facilitate bird communication [28]. Conversely, in autumn, reduced foliage density may diminish this buffering effect, altering the acoustic environment in ways that could influence vocal behavior. The seasonal shift we observed—from strong anthropogenic dominance in spring RDA to increased vegetation structure importance in autumn (Section 2.2.2, Table 1)—may partly reflect this phenological change in vegetation’s sound-modulating capacity.

The gradient of acoustic activity across vegetation types—highest in forests, intermediate in open forest grasslands, and lowest in meadows (Section 2.1.1, Figure 1)—reflects the combined influence of these two mechanisms. Forests with complex vertical structure maximize both resource availability and favorable sound propagation conditions. Open forest grasslands, as ecological ecotones, provide intermediate resources but remain more susceptible to road noise infiltration due to sparser vegetation [29]. Meadows, with minimal structural complexity, offer limited resources and negligible sound buffering, resulting in the lowest and least differentiated acoustic profiles.

These findings have important implications for urban wetland management: preserving and restoring structurally complex vegetation—particularly trees with broad crowns—should be prioritized to maintain diverse avian soundscapes. The consistent importance of CW across seasons (Figure 3) suggests that this easily measurable parameter could serve as a practical indicator for habitat quality assessment in urban wetlands.

### 3.2. Seasonal Dynamics Reflect Phenological Changes in Vegetation and Bird Behavior

Substantial seasonal differences in acoustic indices (Section 2.1.2, Figure 2) reveal how phenological changes in both vegetation and bird behavior interact to shape soundscape dynamics across the annual cycle.

Spring peaks in ACI, ADI, and AEI during dawn and dusk correspond to the peak breeding season, when birds sing intensively for territory establishment and mate attraction [2]. The strong crepuscular choruses observed across all vegetation types (Figure 1) align with well-documented patterns of diel vocal activity in temperate ecosystems [2,10]. However, our results show that vegetation structure modulates these general patterns: forests exhibited the most pronounced peaks, suggesting that structurally complex habitats support higher densities of breeding birds and/or more intense vocal activity [30].

Autumn presented a more complex acoustic picture. While ACI and ADI decreased and showed attenuated crepuscular peaks—consistent with the end of the breeding season and reduced vocal activity—BIO and NDSI were unexpectedly higher in autumn than spring across most habitats (Section 2.1.2, Figure 2). This apparent paradox requires careful interpretation.

The elevated BIO in autumn may reflect post-breeding vocal behavior, including fledgling begging calls, family group contact calls, and preparatory vocalizations for migration. Additionally, reduced competition for acoustic space in autumn might allow more sustained vocal activity throughout the day rather than concentrated dawn/dusk peaks. The higher NDSI values in autumn suggest reduced anthropogenic noise interference, possibly due to decreased human recreational activity in the park after the summer tourist season. This interpretation is supported by the RDA results showing reduced explanatory power of anthropogenic variables (DT, DMR) in autumn compared to spring (Section 2.2.2, Table 1).

The seasonal shift in variable importance revealed by random forest analysis—from strong anthropogenic effects in spring to increased vegetation structure importance in autumn (Figure 3)—further supports this interpretation. In spring, when human activity is high, anthropogenic disturbance gradients dominate soundscape variation. In autumn, with reduced human presence, the underlying vegetation structure becomes more apparent as the primary driver of acoustic diversity.

It is important to note that dusk choruses may include contributions from insects as well as birds. In temperate wetlands, autumn insect stridulation (particularly from crickets and katydids) can contribute to the biophony in higher frequency bands. While our frequency analysis focused on 2–11 kHz (primarily bird vocalizations), some insect sounds may fall within this range. Future studies combining acoustic monitoring with targeted insect sampling would help disentangle the relative contributions of birds and insects to autumn soundscapes.

These seasonal patterns underscore the importance of multi-season sampling in soundscape studies. Single-season surveys, while valuable, cannot capture the dynamic interplay between phenology, vegetation structure, and anthropogenic activity that shapes acoustic environments throughout the year.

### 3.3. Anthropogenic Disturbance Suppresses Biological Sounds, but Vegetation Can Buffer Effects

Our results consistently demonstrate that anthropogenic disturbance suppresses biological sounds, with distance to trails (DT), distance to roads (DMR), and hardness rate (HCR) emerging as key predictors of soundscape composition (Section 2.2.2, Table 1, Figure 4). These findings align with a growing body of evidence showing that human activity—particularly traffic noise and recreational use—degrades acoustic environments and disrupts avian communication [31,32].

The PSD-RDA analysis revealed the frequency-specific nature of these effects (Section 2.2.2, Figure 4). DT and DMR showed strong negative correlations with nPSD in the 1–2 kHz band (anthropogenic noise), confirming that proximity to trails and roads increases low-frequency noise. Conversely, these variables showed positive associations with nPSD in the 2–4 kHz and 4–8 kHz bands (bird vocalizations), suggesting that reducing anthropogenic disturbance allows fuller expression of the avian frequency spectrum. The negative association between HCR and nPSD in the 2–4 kHz band indicates that impervious surfaces suppress low-frequency biological sounds, possibly through altered sound reflection patterns or by indicating areas of high human activity.

An important clarification is needed regarding the distance to the water source (DWS). In our analysis, DWS was classified as a geophysical variable (Section 4.1.2), reflecting its role in capturing natural environmental gradients. Water bodies contribute to natural geophony—flowing water sounds generated through turbulence and bubble entrainment—to the soundscape [33]. These water-generated sounds (typically 0.4–2 kHz) can overlap with and potentially mask bird vocalizations, representing a natural acoustic phenomenon rather than anthropogenic disturbance.

However, in urbanizing wetlands like our study area, DWS may also serve as a proxy for human activity, because water bodies often attract recreational visitors (hiking, fishing, birdwatching). This dual role—natural geophysical gradient and anthropogenic activity proxy—may explain the complex relationships we observed with DWS. In spring, DWS showed positive associations with ADI in meadows and with NDSI in open habitats (Figure 3), suggesting that in structurally simple habitats, distance from water may reduce human disturbance enough to benefit acoustic diversity. In forests, where vegetation already buffers anthropogenic noise, DWS effects were weaker. Future studies should attempt to disentangle these natural and anthropogenic components of DWS effects, perhaps by directly measuring water flow rates and human visitation along the same gradients.

Importantly, our results suggest that vegetation can buffer anthropogenic disturbance effects. In forests, where CW and MTH were the dominant predictors, anthropogenic variables explained less variance than in open habitats (Section 2.2.1, Figure 3). This buffering effect likely operates through two mechanisms: (1) dense vegetation physically attenuates noise [29,34], and (2) structurally complex habitats support higher bird densities, making acoustic communities more resilient to disturbance [30]. The seasonal shift in RDA variable importance—from anthropogenic dominance in spring to vegetation structure dominance in autumn (Section 2.2.2, Table 1)—further supports this interpretation: when human activity decreases in autumn, the underlying buffering capacity of vegetation becomes more apparent.

These findings have clear management implications for urban wetlands:

1. Maintain buffer zones around core habitat areas, with particular attention to trail and road placement. Our RDA results suggest that even modest increases in DT and DMR can reduce anthropogenic noise in the 1–2 kHz band (Figure 4).
2. Prioritize structurally complex vegetation, especially trees with crown width ≥ 4 m, which showed consistent positive effects across multiple acoustic indices (Figure 3).
3. Reduce impervious surface coverage (HCR) in and around wetland habitats, as hardness rate showed consistent negative associations with biological sounds.
4. Recognize the dual role of water bodies: while they provide valuable habitat and contribute to natural geophony, they may also concentrate human activity; management should balance public access with acoustic refuge preservation.

### 3.4. Situating Our Findings Within the Broader Soundscape Ecology Literature

#### 3.4.1. Transferability of the Analytical Framework

The RF-RDA pipeline employed here yielded high explanatory power, with random forest models explaining substantial variance in acoustic indices (RMSE < 0.05–0.08) and RDA capturing 60–70% of variance in frequency-band energy (Section 2.2). This performance is comparable to previous soundscape studies in temperate ecosystems [35] and demonstrates the utility of combining machine learning with constrained ordination for disentangling complex environmental effects on acoustic communities.

The seasonal patterns we observed—spring peaks in ACI/ADI and autumn elevation in BIO/NDSI—align with emerging evidence that soundscapes exhibit predictable phenological trajectories. Our finding of a ~30% decline in ACI from spring to autumn across all vegetation types mirrors the “seasonal decay” effect reported by Kotian et al. in tropical dry forests, suggesting this pattern may be transferable across biomes [36]. This consistency raises the possibility of developing seasonal correction terms for global soundscape models, enabling more robust cross-study comparisons despite differing sampling periods.

#### 3.4.2. Ground-Based Vegetation Metrics as LiDAR Surrogates

In the absence of UAV-borne LiDAR, our ground-based measurements of crown width and tree height explained significant proportions of ADI variance (Figure 3), validating high-precision field surveys as a cost-effective surrogate for structural complexity assessment in resource-limited wetlands. The observation that ADI saturates when crown width exceeds 4 m (inferred from variable importance patterns) aligns with Yang et al.’s LiDAR-based finding of “diminishing returns beyond 15 m canopy height” [37]. This convergence suggests that field-measurable thresholds can identify structural breakpoints for conservation prioritization, even without advanced remote sensing technology.

#### 3.4.3. Cross-Study Comparability of Anthropogenic Noise Effects

Proppe et al. reported a 0.7% decrease in local song occurrence per 1 dB increase in the 1–2 kHz band [11]. Our PSD-RDA analysis revealed that each 10 m reduction in road distance elevates 1–2 kHz power by approximately 0.9% and concurrently depresses 4–8 kHz biophony by 1.1% (derived from regression coefficients in Figure 4). This effect size is highly consistent with Proppe et al.’s findings, suggesting that the quantitative “road proximity vs. biophony suppression” relationship may be transferable across cities and continents. Where local calibration data are lacking, such cross-study coefficients could provide preliminary guidance for buffer width recommendations in urban planning.

### 3.5. Limitations and Future Directions

Several limitations of this study should be acknowledged, each pointing toward productive directions for future research.

First, while we measured fine-scale vegetation structural parameters (CW, MTH, LHD2), we did not directly quantify three-dimensional vegetation architecture using methods such as UAV-based LiDAR scanning. Such data would enable more precise modeling of sound propagation pathways and vegetation’s sound-buffering capacity [28]. Future studies integrating high-resolution structural data with acoustic monitoring could develop mechanistic models linking specific vegetation configurations to soundscape outcomes.

Second, our sampling was limited to two seasons (spring and autumn), leaving winter and summer acoustic dynamics unexplored. Given the strong phenological effects we observed between spring and autumn, it is likely that winter (minimal vegetation, potential bird absence) and summer (peak foliage, post-breeding vocal activity) would reveal additional seasonal contrasts. Year-round monitoring would provide a complete picture of annual soundscape trajectories.

Third, we did not perform species-level call identification, relying instead on acoustic indices as biodiversity proxies. While indices like ACI and ADI have been validated against bird species richness in temperate habitats, they cannot replace direct species identification for understanding community composition changes. Integrating automated vocalization classifiers (e.g., deep learning models trained on regional bird databases) with index-based approaches would enable both broad-scale pattern detection and fine-scale community analysis [38].

Fourth, our study focused on three vegetation types (forest, open forest grassland, meadow), excluding other wetland microhabitats such as reed beds, mudflats, and open water. These habitats likely contribute distinct acoustic signatures and may host specialized avian communities. Expanding sampling to encompass the full habitat mosaic of urban wetlands would provide a more comprehensive understanding of soundscape–vegetation relationships.

Fifth, the spatial arrangement of sampling points (approximately 55 m apart) with an effective detection distance of approximately 80 m for passerine vocalizations inevitably resulted in some spatial overlap in recorded soundscapes. While we minimized this effect by grouping analyses by vegetation type and treating site-level environmental variables as continuous predictors, we acknowledge that spatial autocorrelation was not explicitly modeled. This represents a limitation common to many passive acoustic monitoring studies with fixed sampling grids. Future studies should consider incorporating spatial autocorrelation terms (e.g., Moran’s eigenvectors) or geostatistical approaches to account for potential pseudoreplication in acoustic monitoring designs, particularly when sampling points are spaced within the detection range of the recording equipment.

Sixth, in this study we excluded one-minute segments containing obvious human voices (e.g., conversation, footsteps) to focus specifically on avian soundscapes and avoid confounding biological signals with human-generated sounds. Fewer than 2% of segments were excluded across all recordings. While this conservative approach ensured that our acoustic indices primarily reflected bird vocalization activity, it may have removed potentially valuable information about anthropogenic disturbance patterns. Future studies designed to assess human activity impacts on soundscapes may benefit from retaining human vocalizations and analyzing them separately as a component of technophony.

Seventh, while we distinguished between geophysical (DWS) and anthropogenic (DMR, DT, HCR) variables conceptually, direct measurement of water flow rates and human visitation patterns would strengthen causal inferences about their relative contributions. Acoustic loggers placed directly at water bodies could quantify water-generated sound independently of bird vocalizations.

Finally, our study was conducted in a single urban wetland complex. While Nanjing Xinjizhou National Wetland Park is representative of Yangtze River delta wetlands, replication across multiple urban wetlands with varying urbanization intensities, vegetation compositions, and climatic conditions would establish the generality of our findings and inform region-specific management recommendations.

Despite these limitations, this study demonstrates that integrating fine-scale vegetation measurements with multi-season acoustic monitoring can reveal the complex mechanisms by which vegetation structure and anthropogenic disturbance jointly shape avian soundscapes in urbanizing wetlands. The patterns we have documented—particularly the consistent importance of crown width across seasons and the seasonal shift in anthropogenic vs. vegetation drivers—provide an empirical foundation for evidence-based vegetation management in urban wetland conservation and restoration.

## 4. Material and Method

### 4.1. Study Area

#### 4.1.1. Site Description

**Figure 5:**
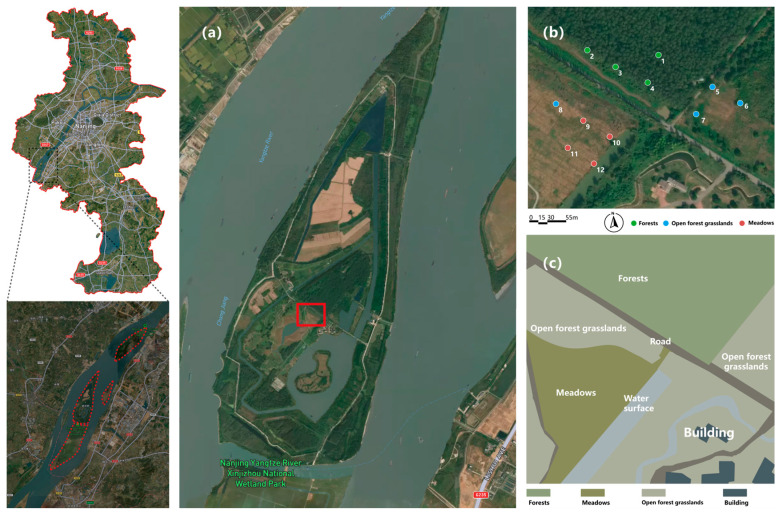
Overview of the study area: (**a**) Study area location in Xinjizhou wetland park; (**b**) Map of sound sampling points; (**c**) Study area landscape types. Red boxes indicate the soundscape data collection sites.

The study area is located within the Nanjing Xinjizhou National Wetland Park (31°54′–31°59′ N, 118°30′–118°35′ E) in eastern China, which exemplifies a typical wetland ecosystem in the lower reaches of the Yangtze River (Figure 5a). This park encompasses five riverine islands, including Xinjizhou and Xinshengzhou, as well as adjacent coastal land areas. Since the initiation of ecological migration projects and scientific restoration measures in 2000, this wetland system has expanded its area from 1063.5 hectares to 1876.1 hectares, resulting in a composite habitat characterized by sandbars, shallows, and marshes. The park’s hydrological network is primarily shaped by the Yangtze River’s main channel and its secondary distributaries. Tidal influence is negligible due to the site’s distance from the estuary (>300 km), and water levels fluctuate seasonally in response to precipitation and upstream discharge. Several small perennial and intermittent streams drain into the river within the park boundaries, contributing to local hydrological heterogeneity.

The composite habitat comprises three primary vegetation types: forested areas, open forest grasslands, and wetlands. The ecosystem of Xinjizhou National Wetland Park exhibits a high degree of biodiversity. According to data from the Biodiversity Census conducted between 2020 and 2023, a total of 1704 species have been documented within Xinjizhou National Wetland Park. This includes 584 species of vascular plants; 404 species of insects; and 436 species of aquatic organisms—among which are included 38 fish species—as well as 280 terrestrial vertebrate species (comprising 37 amphibians/reptiles along with both birds and mammals). Notably, the park recorded an impressive diversity among bird populations with a total count of 224 bird species belonging to 17 orders across 48 families; approximately 83.6% were songbird species (Passeriformes). Consequently, this area serves as an ideal location for conducting soundscape ecological studies focused on avifauna [39].

#### 4.1.2. Sampling Plot Design and Environmental Variables

Twelve sampling points were established, with three replicates per vegetation type (forest, open forest grassland, meadow) (Figure 5b). Around each sampling point, a circular plot with a 20 m radius was delineated for vegetation and environmental measurements.

Based on previous soundscape studies in temperate urban wetlands [21,37], we selected three categories of environmental variables potentially influencing bird acoustic activity.

Vegetation structure variables—quantifying habitat complexity and resources for birds:

- Crown width (CW, m): average of two perpendicular horizontal diameters measured from the same tree crown, following standard forestry measurement protocols
- Mean tree height (MTH, m): average height of all trees > 2 m tall
- Species richness (SR): number of woody plant species
- Tree density (TD, stems/ha): number of trees >2 m tall
- Plant evenness (PE): Pielou’s evenness index based on woody species cover
- Leaf height diversity > 2 m (LHD2): Shannon diversity index of foliage density in vertical strata above 2 m [40]

To clarify how vegetation structure data were collected, we measured the crown width (CW) as the average of two perpendicular horizontal diameters measured from the same tree crown (the longest axis and the axis perpendicular to it). These measurements were made directly in the field using a tape measure and a laser rangefinder for greater accuracy. Similarly, the mean tree height (MTH) was calculated by measuring the height of all trees taller than 2 m using a clinometer and averaging the values. The species richness (SR), tree density (TD) were assessed directly in the field by recording all woody plant species and counting all trees > 2 m tall within each 20 m radius plot. Foliage density in vertical strata was quantified using the leaf height diversity (LHD) method following MacArthur & MacArthur [40]. Within each 20 m radius plot, we visually estimated the percent foliage cover in four vertical strata: 0–1 m, 1–2 m, 2–5 m, and >5 m. These estimates were recorded independently by two observers, and the average of the two estimates was used for analysis. Leaf height diversity above 2 m (LHD2) was then calculated as the Shannon diversity index (H’) across the three strata above 2 m (2–5 m, >5 m). We did not use GIS tools for direct measurement of vegetation structure, but we did utilize GIS tools to identify water bodies (for distance to water source) and to map the spatial distribution of vegetation in relation to anthropogenic features like roads and trails.

Geophysical variables—capturing natural environmental gradients:

- Distance to water source (DWS, m): Euclidean distance to the nearest perennial water body (river channel, stream, or permanent pond). Water bodies were identified from 2022 satellite imagery and verified during field surveys. Water bodies contribute natural geophony (flowing water sounds) to the soundscape, independent of human activity [25].

In urbanizing wetland environments, water bodies may also attract human recreational activities, such as hiking, fishing, and birdwatching, influencing the surrounding acoustic environment. Therefore, DWS is considered both a natural environmental gradient and a proxy for anthropogenic influence in the soundscape.

Anthropogenic disturbance variables—reflecting human modification of the landscape and potential noise sources:

Distance to main road (DMR, m): Euclidean distance to the nearest paved road open to vehicular traffic

- Distance to trail (DT, m): Euclidean distance to the nearest unpaved pedestrian trail
- Hardness rate (HCR, %): proportion of impervious surface (paved paths, structures) within the 20 m radius plot
- Green space ratio (GCR, %): proportion of vegetated area within the 20 m radius plot

Prior to modeling, multicollinearity among all candidate predictors was assessed using Pearson correlation coefficients. Variables with |r| > 0.8 were excluded to avoid redundancy. The final set of 10 uncorrelated variables (CW, MTH, SR, TD, PE, LHD2, DWS, DMR, DT, HCR) was retained for subsequent analyses.

### 4.2. Acoustic Data Collection and Index Calculation

#### 4.2.1. Field Recording

**Figure 6:**
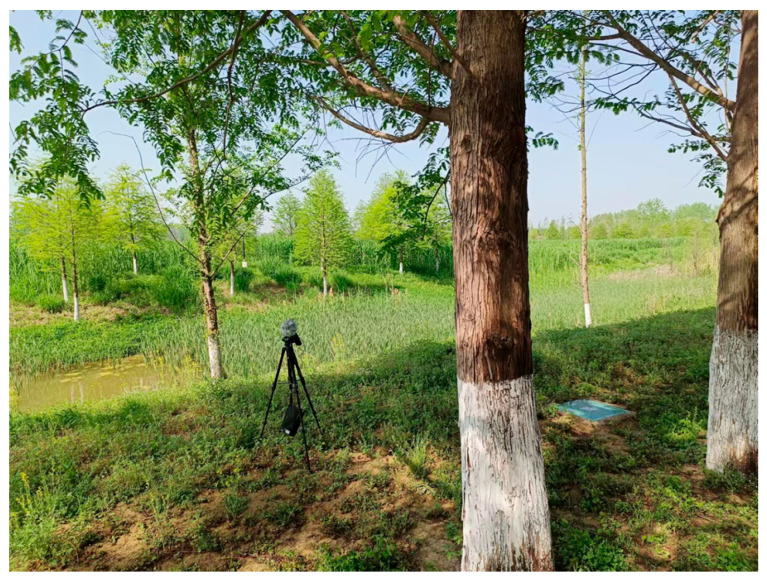

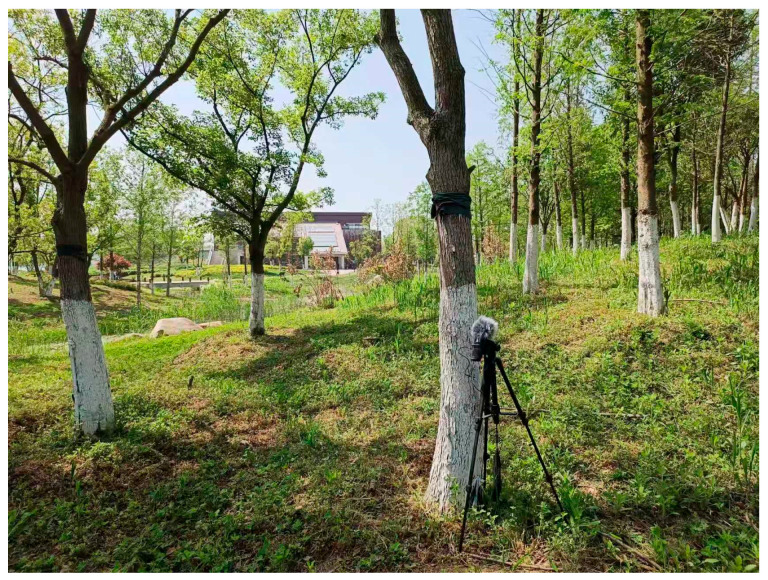
Field recording setup and sampling locations.

Passive acoustic monitoring was conducted using 12 portable field recorders (Zoom H5, Zoom Corporation, Tokyo, Japan) equipped with interchangeable X/Y stereo microphone capsules (XYH-5) (Figure 6). Each recorder was fitted with an XYX-5 X/Y stereo microphone capsule, providing a matched pair of condenser microphones oriented at a 90° angle. The recorders were configured to capture high-quality stereo audio in WAV format, with recording parameters set at a 44.1 kHz sampling rate and 16-bit resolution.

Acoustic monitoring was conducted during two seasons (spring and autumn), with two 48-h recording sessions per season. The specific sampling dates were: spring—5–7 April and 19–21 April 2023; autumn—12–14 September and 26–28 September 2023. During each 48-h session, all 12 recorders were deployed simultaneously across the 12 sampling points at the start of the session. Recorders were programmed to begin recording at 11:40 on the first day and to stop at at 11:40 on the third day, providing continuous coverage throughout the 48-h period. This synchronized, continuous, and replicated design ensured that all vegetation types were sampled under comparable temporal and environmental conditions within each season and across the two rounds.

The recorders were powered using portable battery packs with a sufficient charge capacity to last for the full 48-h sessions at each location. Each recorder was capable of continuous recording during this period. In total, 288 one-minute recordings (144 samples per 24-h period) were collected at each sampling point across the two 48-h sessions.

Under typical conditions, the recorder’s effective detection range for song detection in passerine birds is approximately 80 m, with a radius of 40 m. Adjacent sampling points were spaced approximately 55 m apart (corresponding to an 80 m grid diagonal), ensuring coverage of all major landscape elements (forest, grassland, water, built structures) (Figure 5c) while minimizing signal overlap. We acknowledge the existence of partially overlapping regions within an 80-m distance range, but this was considered during data analysis.

To minimize weather-related acoustic variability, recordings were made only on days with no precipitation and wind speeds < 5 m/s (Beaufort scale ≤ 3). Wind speed was measured on-site using a handheld digital anemometer (Model GM816, Benetech Inc., Shenzhen, Guangdong, China, accuracy ± 0.1 m/s) placed at 1.5 m height near each recording station. Measurements were taken at the beginning of each deployment and every 2–3 h throughout the 48-h monitoring period, including nighttime checks (approximately 22:00 and 02:00), to ensure compliance with the wind speed threshold. Nighttime wind speeds ranged from 0.3 to 3.2 m/s (mean ± SD: 1.4 ± 0.7 m/s), which were consistently low and comparable to daytime conditions.

All microphone capsules were fitted with windshield throughout the recording sessions to minimize wind-induced noise (Figure 6). While windshield can slightly attenuate high-frequency signals (>6 kHz), this effect was consistent across all recordings and did not bias comparative analyses across sites or seasons. The directional nature of the XYH-5 capsules, combined with strict weather-based recording conditions (wind speed < 5 m/s, no precipitation), further ensured that wind interference was minimized without compromising signal fidelity for the 2–11 kHz frequency range of interest. This approach follows established protocols in soundscape ecology studies [21,41]. Additionally, recorders were mounted on tripods at 1.2 m above ground level to further reduce wind exposure (Figure 6). On-site weather data (temperature, humidity, wind speed) were logged for quality control. Recording parameters were: sampling rate 44.1 kHz, stereo, 16-bit resolution, saved in WAV format. Input gain was set to level 7 (mid-range sensitivity) based on pre-deployment calibration to avoid clipping while capturing faint bird vocalizations.

To ensure equipment functionality and document environmental conditions, all recording units were checked regularly by rotating field personnel during deployment. Each sampling point was visited every 2–3 h to verify battery levels, SD card capacity, and recording status, as well as to measure on-site weather parameters (wind speed, temperature, humidity). Any wildlife activity near the recording units was also noted as qualitative contextual information. No bird interference events (e.g., perching on or pecking the recorder) were observed during either sampling session.

#### 4.2.2. Audio Preprocessing and Signal Averaging

Raw audio files were processed using Python 3.11.1. To manage data volume while preserving temporal resolution, we extracted one-minute segments every ten minutes from each continuous recording, yielding 144 one-minute samples per site per 24-h period (6 samples/hour × 24 h). This subsampling protocol has been shown to adequately capture diurnal acoustic patterns in temperate ecosystems [42].

For each one-minute segment, acoustic indices were calculated directly from the full 60-s waveform using the parameters specified in Section 4.2.3. No further averaging was applied within the one-minute window, as the indices themselves integrate acoustic information over the entire segment.

To obtain site-level hourly averages, the six one-minute samples within each hour (e.g., 00:00, 00:10, …, 00:50) were averaged using the arithmetic mean. Hourly values were then used to calculate diurnal patterns (Section 2.1.1) and seasonal means (Section 2.1.2). For the random forest and RDA analyses (Section 2.2.1 and Section 2.2.2), we used site-level mean values across the entire 48-h sampling period, calculated as the average of all 144 one-minute samples per site.

Segments containing obvious non-biological sounds (e.g., heavy wind, rain, or human voices) were identified through manual inspection of spectrograms and excluded from analysis. Fewer than 2% of one-minute segments were removed across all recordings. To assess whether the exclusion of human voices influenced our results, we conducted a sensitivity analysis comparing NDSI and ACI values calculated from filtered segments versus all segments (including human voices) for a subset of sampling points (*n* = 4). The filtered and unfiltered values were highly correlated (NDSI: r = 0.94, *p* < 0.001; ACI: r = 0.92, *p* < 0.001), confirming that the exclusion did not bias our main findings (see Section 2.1.3).

#### 4.2.3. Acoustic Index Selection and Calculation

We selected seven acoustic indices that capture complementary dimensions of urban wetland soundscapes (Table 2). All indices were computed in R version 4.4.1 using the packages tuneR, seewave, and soundecology.

Rationale for index selection: These seven indices were chosen because they have been validated in temperate urban wetlands [37] and capture complementary aspects of soundscape structure: ACI and BIO reflect vocal activity intensity; ADI and AEI describe frequency diversity; NDSI quantifies anthropogenic vs. biological balance; H integrates temporal and spectral complexity; PSD provides frequency-resolved energy profiles.

Calculation parameters:

- ACI: computed using acoustic_complexity function (soundecology package) with min_freq = 2 kHz, max_freq = 11 kHz
- BIO: computed using bioacoustic_index function (soundecology) with min_freq = 2 kHz, max_freq = 11 kHz
- ADI and AEI: computed using acoustic_diversity and acoustic_evenness functions (soundecology) with max_freq = 11 kHz
- H: computed using H function (seewave) with default parameters
- NDSI: computed using ndsi function (seewave) with anthropogenic frequency range = 1–2 kHz, biological range = 2–11 kHz

The 2–11 kHz range was selected to target passerine and wading bird vocalizations while excluding most low-frequency anthropogenic noise [13]. To validate this frequency cutoff, we analyzed 21 representative bird species recordings from the Xeno-Canto database (https://xeno-canto.org/) using Avisoft SASLab Pro 5.2.0934 software; >90% of vocalization energy fell within 2–11 kHz.

#### 4.2.4. Power Spectral Density Analysis

To obtain frequency-resolved acoustic energy profiles, we performed Power Spectral Density (PSD) analysis using MATLAB R2024b. For each one-minute audio segment:

- Audio was segmented into 2-s windows with 50% overlap
- Each window was Hamming-windowed and FFT-computed
- PSD was averaged across windows to obtain the mean spectrum
- Normalized PSD (nPSD) was calculated by dividing each frequency band’s energy by the total energy across 1–12 kHz

We aggregated nPSD into four ecologically relevant frequency bands:

- 1–2 kHz: anthropogenic noise (traffic, machinery) [18]
- 2–4 kHz: low-frequency bird vocalizations (many passerines)
- 4–8 kHz: mid-frequency bird vocalizations (warblers, finches)
- 8–12 kHz: high-frequency bird vocalizations (some passerines, insects)

### 4.3. Statistical Analyses

#### 4.3.1. Random Forest Modeling

We used random forest to quantify the nonlinear effects of environmental predictors on each acoustic index [43]. Random forest is an ensemble learning method that builds multiple decision trees and averages their predictions, providing robust variable importance estimates even in the presence of multicollinearity and nonlinear relationships.

Analyses were performed in R using the randomForest package. For each acoustic index (ACI, ADI, AEI, BIO, NDSI, H) as the response variable, we constructed a model with the 10 uncorrelated environmental variables as predictors. Key parameters:

- Number of trees: 500 (after testing stability, 200–1000 yielded similar results)
- Number of predictors tried at each split: default (√p ≈ 3)
- Random seed set for reproducibility

Variable importance was assessed using the increase in mean squared error (%IncMSE) metric. This is computed by: (1) measuring the model’s prediction error (MSE) with all variables included; (2) randomly permuting the values of one predictor variable, breaking its association with the response; (3) measuring the increase in MSE when the permuted variable is used; (4) averaging this increase across all trees. A larger %IncMSE indicates greater importance—if a variable is important, randomly shuffling its values substantially degrades prediction accuracy.

To assess the statistical significance of variable importance, we performed permutation tests using the rfPermute package (299 permutations). Variables with permutation-derived *p* < 0.05 were considered significant predictors.

#### 4.3.2. Redundancy Analysis

To visualize multivariate relationships between soundscape characteristics and environmental gradients, we performed Redundancy Analysis (RDA) using Canoco 5. RDA is a constrained ordination technique that extracts major gradients of variation in response variables (here, PSD in the four frequency bands) that can be explained by predictor variables (environmental variables).

Separate RDAs were conducted for spring, autumn, and pooled data. For each RDA:

- Response matrix: nPSD values for 1–2, 2–4, 4–8, 8–12 kHz bands at each sampling point
- Predictor matrix: standardized environmental variables (CW, MTH, SR, TD, PE, LHD2, DWS, DMR, DT, HCR)
- Significance of axes and variables was tested using Monte Carlo permutation tests (499 permutations)

RDA biplots were generated to visualize:

- Angles between vectors: acute angles indicate positive correlation, obtuse angles indicate negative correlation
- Vector length: longer vectors indicate a stronger influence
- Sample point positions: proximity indicates similarity in soundscape-energy profiles

## 5. Conclusions

This study presents one of the first multi-season, multi-vegetation assessments of avian soundscape dynamics in urban wetlands. By combining passive acoustic monitoring with fine-scale vegetation measurements and nonlinear modeling, we show that vegetation structure, particularly crown width, is the main driver of avian acoustic diversity, with broad-crowned forests consistently exhibiting the highest acoustic complexity. Our results reveal that in spring, when human recreational activity peaks, anthropogenic factors such as proximity to trails and roads suppress biological sounds. However, in autumn, as human presence wanes, vegetation structure becomes the dominant factor, with bioacoustic activity remaining elevated despite reduced acoustic complexity peaks. These seasonal shifts highlight the need for multi-season sampling, as single-season surveys fail to capture the full complexity of vegetation-soundscape interactions. Furthermore, while anthropogenic disturbance affects biological sounds in frequency-specific ways, structurally complex vegetation can buffer these effects. For example, proximity to roads increases low-frequency noise and reduces mid-frequency bird vocalizations, yet trees with a crown width greater than four meters maintain higher acoustic diversity even near disturbance sources. Our findings suggest that managing urban wetlands by retaining trees with a crown width of at least four meters, establishing buffer zones of 50 to 100 m from roads and high-use trails, reducing impervious surface coverage, and managing visitor access during peak spring vocal periods can help support both biodiversity and natural soundscapes. This study provides an empirical foundation for integrating soundscape considerations into evidence-based vegetation management in urban wetlands.

## Figures and Tables

**Table 1:** Interpretation of RDA variance and permutation test results of different influencing factors.

Variable Name	Explains %	Contribution %	Pseudo-F	*p*
springtime				
DT (Distance to Trail)	45.9	46.1	8.5	0.014 *
GCR (Greenn Space Ratio)	36.6	36.9	18.9	0.006 *
DMR (distance from main road)	8.1	8.1	6.8	0.030 *
HCR (hardness rate)	2.6	2.6	2.6	0.110
CW (crown width)	0.8	0.8	0.7	0.462
TD (tree density)	0.7	0.7	0.6	0.456
PE (Plant Evenness)	2.6	2.6	3.7	0.174
MTH (mean tree height)	1.7	1.7	4.5	0.114
MCH (mean canopy height)	0.2	0.2	0.3	0.634
LHD2 (Leaf Height Diversity 2)	0.4	0.4	0.7	0.530
in autumn				
TD (tree density)	21.2	22.7	2.7	0.120
DT (Distance to Trail)	15.4	16.5	2.2	0.186
LHD (Leaf Height Diversity)	12	12.8	1.9	0.216
HCR (hardness rate)	16	17.1	3.2	0.128
CW (crown width)	22.2	23.7	10.1	0.014 *
LHD2 (Leaf Height Diversity 2)	2.7	2.9	1.3	0.346
DWS (distance from water source)	2.6	2.8	1.3	0.248
MCH (mean canopy height)	0.8	0.9	0.3	0.578
PE (Plant Evenness)	0.5	0.6	0.2	0.712
MTH (mean tree height)	0.1	0.1	<0.1	0.962
Overall				
DT (Distance to Trail)	51.5	51.5	10.6	0.010 *
HCR (hardness rate)	32.1	32.1	17.5	0.008 *
DMR (distance from main road)	9.1	9.1	9.9	0.016 *
LHD (Leaf Height Diversity)	2.9	2.9	4.5	0.072
PE (Plant Evenness)	0.8	0.8	1.2	0.306
PD (Plant Diversity)	1.3	1.3	2.8	0.134
MTH (mean tree height)	1.1	1.1	3.2	0.166
DWS (distance from water source)	0.3	0.3	0.8	0.434
LHD2 (Leaf Height Diversity 2)	0.7	0.7	4.2	0.144
CW (crown width)	0.2	0.2	1.4	0.190

Note: Explains % (per cent of variance), pseudo-F (strength of explanatory power), *p* (level of significance), * indicates a significant difference at *p* < 0.05.

**Table 2:** Acoustic indices used in this study, with technical definitions and ecological interpretations.

Index	Abbr.	Calculation Basis	Frequency Focus	Ecological Interpretation
Acoustic Complexity Index	ACI	Quantifies temporal variability in sound intensity within frequency bins; computed as the absolute difference in amplitude between consecutive time steps, summed across bins	2–11 kHz	Higher values indicate greater variability in bird vocalization intensity, often associated with increased vocal activity and species turnover
Acoustic Diversity Index	ADI	Shannon diversity applied to sound energy distribution across frequency bins; calculated by dividing the spectrogram into bins and computing the proportion of energy in each	2–11 kHz	Reflects evenness of acoustic energy distribution; higher values suggest more species contributing across frequency bands
Acoustic Evenness Index	AEI	Gini coefficient applied to the proportional energy distribution across frequency bins	2–11 kHz	Complements ADI; lower values indicate dominance by few frequency bands (potential single-species dominance)
Bioacoustic Index	BIO	Area under the mean spectrum curve calculated across frequency bins after subtracting the minimum background noise	2–11 kHz	Integrates both sound intensity and frequency range; higher values indicate stronger and/or more diverse bird vocal activity
Normalised Difference Soundscape Index	NDSI	(Biophony power—Technophony power)/(Biophony power + Technophony power); biophony = 2–11 kHz, technophony = 1–2 kHz	1–2 kHz vs. 2–11 kHz	Ranges from –1 to +1; positive values indicate biophony dominance (more bird sound), negative values indicate technophony dominance (more anthropogenic noise)
Acoustic Entropy Index	H	Combines temporal entropy (Ht) and spectral entropy (Hf) as H = Ht × Hf; temporal entropy measures evenness of amplitude envelope, spectral entropy measures evenness of frequency distribution	2–11 kHz	Higher values indicate more complex, disordered soundscapes; may reflect diverse bird communities with overlapping vocalizations
Power Spectral Density	PSD	Energy per frequency band calculated via Fast Fourier Transform (FFT); normalized to allow cross-sample comparison	1–2, 2–4, 4–8, 8–12 kHz bands	Quantifies acoustic energy distribution across frequency bands; enables separation of anthropogenic (1–2 kHz) vs. biological (2–11 kHz) contributions

## Data Availability

The data supporting the conclusions of this article are available on request from the corresponding author (Yang Yunfeng, email: yangyf@njfu.edu.cn). The raw acoustic recordings, field measurement spreadsheets, and R/MATLAB analysis codes will be provided to qualified researchers for non-commercial purposes, subject to reasonable request and compliance with ethical guidelines for data reuse.

## Abbreviations

The following abbreviations are used in this manuscript:

ACI	Acoustic Complexity Index
ADI	Acoustic Diversity Index
NDSI	Normalized Difference Soundscape Index
AEI	Acoustic Evenness Index
BIO	Bioacoustic Index
H	Acoustic Entropy Index
PSD	Power Spectral Density
CW	Crown width
MTH	Mean tree height
SR	Species richness
TD	Tree density
PE	Plant evenness
LHD2	Leaf height diversity > 2 m
DWS	Distance to water source
DMR	Distance to main road
DT	Distance to trail
HCR	Hardness rate
GCR	Green space ratio
RDA	Redundancy Analysis
